# COVID-Related Athletic Deaths: Another Perfect Storm?

**DOI:** 10.3389/fspor.2022.829093

**Published:** 2022-04-12

**Authors:** Philip B. Maffetone, Paul B. Laursen

**Affiliations:** ^1^Independent Researcher, Storrs, CT, United States; ^2^Sport Performance Research Institute New Zealand (SPRINZ), Auckland University of Technology, Auckland, New Zealand

**Keywords:** COVID-19, vaccine, sudden athlete cardiac death, inflammation, nutrition, high intensity (strenuous) exercise

## Introduction

Despite high cardiorespiratory fitness, athletes of all ages and sex can suffer poor health, including cardiac conditions; some may even die during training or competition. While athletes are often thought of as being very healthy, this is not always the case as many are fit but unhealthy (Maffetone and Laursen, 2015; Scudiero et al., 2021). Sudden cardiac death (SCD) is one example.

The causes of SCD in athletes vary, with the estimated incidence of death between 1 in 40,000 to 1 in 80,000 persons (Harmon et al., 2014). The wide range may be due in part to the definitions of SCD; some estimates include only deaths with exertion or shortly (<1 h) after exertion, others include any SCD in an athlete (exertional or outside of exertion) and exclude those who have been resuscitated from sudden cardiac arrest. In athletes <35 years, inherited cardiac conditions such as hypertrophic cardiomyopathy and anomalous origin of a coronary artery are most common. Athletes >35 years represent the most sudden death cases from atherosclerotic coronary artery disease, which are usually lifestyle related (Lechner et al., 2020). The true incidence of SCD may be unknown and underestimated as current estimates are based largely on case identification through public media reports and estimated participation rates (Harmon et al., 2011). In addition, underreporting in all ages may occur in the context of recreational sports (Marijon et al., 2011).

While SCD in athletes is relatively rare, even a single case represents too many, especially considering most are preventable. This article highlights additional risks associated with post-COVID-19 infection and vaccination.

## Myocarditis

Myocarditis is a risk factor for SCD. This non-ischemic inflammatory heart muscle disease can lead to cardiac dysfunction and arrhythmias, often resulting from infectious and/or autoimmune insults, with viral myocarditis in asymptomatic athletes being a common cause, especially in those <35 years (Daniels et al., 2021).

The increased risk of SCD with exercise is associated with accelerated and progressive inflammation (Basso et al., 2007). Findings of myocarditis or myocardial inflammation in asymptomatic or mildly symptomatic competitive athletes and members of the U.S. military during or after COVID-19 infection using cardiac magnetic resonance imaging have been recently described (Daniels et al., 2021; Rajpal et al., 2021; Seeherman and Suzuki, 2021). In a descriptive study of 1,626 cases of myocarditis in a national passive surveillance report in the U.S., rates within 7 days after vaccination exceeded the expected rates across multiple age and sex strata and were highest after the second vaccination dose in adolescent males and in young men (Oster et al., 2022). From a cohort study of 1,597 U.S. university competitive athletes following COVID-19 infection, 37 athletes (2.3%) were diagnosed with clinical and subclinical myocarditis. Indeed, it has been known for some time that physical activity can increase the risk of death in those with myocarditis and other cardiovascular conditions. For example, in an autopsy study of US Air Force recruits with SCD, physical activity was a risk factor with unrecognized myocarditis as the most common suspected underlying factor (Phillips et al., 1986). Numerous other studies demonstrate similar results and show that the prevalence of signs on CMR imaging of myocarditis is in the range of 1–3% in athletes following positive COVID-19 test results (Udelson et al., 2021).

In addition to myocarditis, pericarditis, inflammation of the pericardium, has also been observed in post-COVID infected patients (Brito et al., 2021). In 54 previously healthy college athletes who tested positive for COVID-19, Brito et al. (2021) found that more than a third showed imaging features of pericardial inflammation. In particular, severe cases of myocarditis and pericarditis can result in chronic heart failure or death and are therefore important public health concerns (Husby et al., 2021). While the biological mechanisms are not yet clear, the same adverse events were attributed to use of the smallpox vaccine in adults (Halsell et al., 2003).

## High-Intensity Exercise

High-intensity exercise, a common risk factor for overtraining (Maffetone and Laursen, 2015), can exert immune suppression with even a single bout in certain individuals that can last for several days, increasing the risk of infection (Shephard and Shek, 1994). With non-infectious myocarditis, high-intensity exercise can also trigger an inflammatory response sufficient to promote structural changes in the heart (Hosenpud et al., 1987). A resulting cytokine storm can lead to an excessive and uncontrolled inflammatory response, with an accompanying feedback loop between catecholamines and cytokines, and clinical complications associated with cardiac and respiratory distress, and hypercoagulation (Staedtke et al., 2018; Gill et al., 2022). The myocardial injury observed in the hearts of patients recently vaccinated has a similar histologic appearance as catecholamine-mediated stress cardiomyopathy and severe SARS-COV-2 infection, including “myocarditis” which is associated with cytokine release syndrome (Gubbi et al., 2020). These instances may be different from other more common cases of myocarditis (Gill et al., 2022).

Normally when the SARS-CoV-2 enters the body, its spike protein binds with the angiotensin-converting–enzyme 2 (ACE2) receptor, gaining entry to the cell, triggering an immune response by producing protective antibodies against the virus. Theoretically, immune-mediated side effects of COVID-19 infection, and vaccines which use the same antigen as the SARS-CoV-2 spike protein, could involve the production of secondary anti-idiotype (Ab2) antibodies (Murphy and Longo, 2021). These can bind to and deplete the initial protective antibody responses, mirroring the original antigen, and potentially lead to adverse immune, cardiac, and neurologic side-effects, including long-COVID (Di Toro et al., 2021).

**Figure 1:**
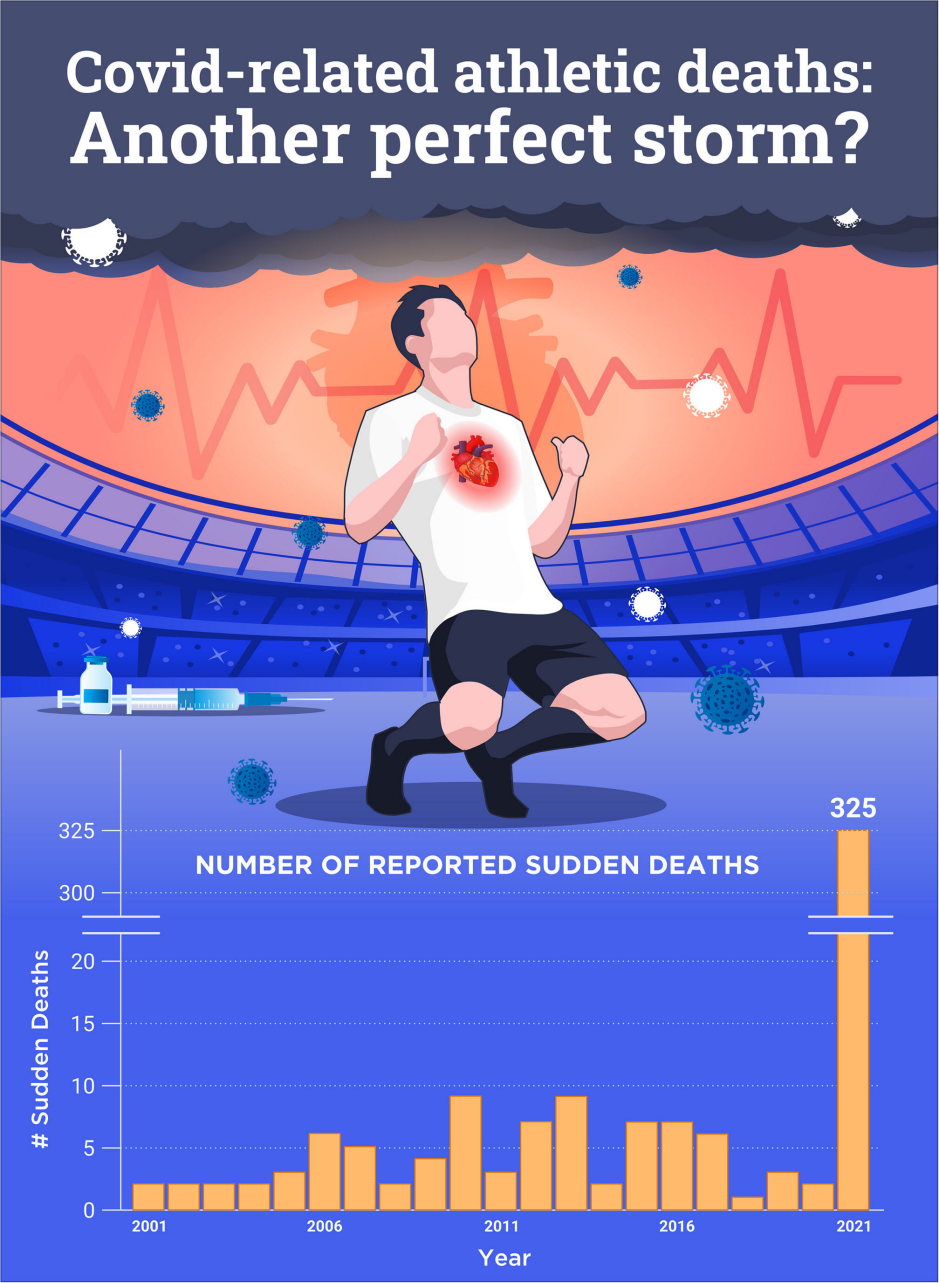
Number of reported sudden cardiac deaths post-COVID-19 and/or vaccination worldwide in 2021 compared to 2001–2020. Ref: https://goodsciencing.com/covid/athletes-suffer-cardiac-arrest-die-after-covid-shot/.

Increased risk of COVID-19 infection includes those with chronic inflammatory-related comorbidities, while the infection itself is associated with acute inflammation and immune impairment, previously described as a perfect storm (Maffetone and Laursen, 2020). Recently, we have witnessed two other closely related conditions that add to the burden of SCD in athletes: COVID-19 and the related vaccinations, and sudden death (Figure 1).

Pharmacovigilance reports, health system surveillance and case studies, and other published studies have suggested an association between SARS-CoV-2 vaccination and myocarditis and pericarditis in the general population (Husby et al., 2021). COVID vaccines can increase inflammation on the endothelium with accompanying T cell infiltration of cardiac muscle and may account for increased thrombosis, cardiomyopathy, and other vascular events following vaccination in athletes (Daniels et al., 2021; Grundy, 2021). The relationship between COVID vaccines and the increased risk of myocarditis was recently addressed by the Centers for Disease Control and Prevention (CDC) in a statement on the potential link between the BNT162b2 and the mRNA-1273 COVID vaccines and myocarditis and pericarditis (Prevention CfDCa, 2021). Researchers in Israel reported that vaccination increased the 42-day risk of myocarditis by a factor of 3.24 [95% confidence interval (CI), 1.55–12.44] as compared with the risk among unvaccinated persons; events that were mostly concentrated among young male patients (Barda et al., 2021). In addition to myocarditis, which tends to develop rapidly in younger patients, mostly after a second vaccination, pericarditis is also observed after COVID-19 vaccination and tends to affect older patients later, after either the first or second dose (Diaz et al., 2021). Most recently, Cadegiani (2022) theorized that the that a “hypercatecholinergic” state, as witnessed in sport and during training, is the key trigger of the heart complications leading to sudden death in athletes with likely undetected myocarditis/pericarditis.

## Prevention and Mitigation Moving Forward

Many athletes may not have clinically apparent signs and symptoms and first present with sudden death, although ~30% of athletes with SCD have been reported to have had symptoms such as chest pain, shortness of breath, performance decline, palpitations, pre-syncope, or syncope leading up to the event (Marijon et al., 2015). This represents the potential for better screening, education, and medical care in preventing such events. The incidence of SCD in competitive athletes can be substantially reduced with preparticipation screening (Corrado et al., 2006; Asif and Drezner, 2016; Mennitti et al., 2020). In addition, strongly recommending the implementation of prevention through healthier lifestyles, especially diet, are known to reduce cardiovascular risk factors in adults (Alvarez-Alvarez et al., 2018). The same should be emphasized in younger athletes as lifestyle improvements can decrease risk for atherosclerosis in middle age (Spring et al., 2014). Moreover, practitioners, coaches, and individuals engaged in sports, vaccinated or not, can help reduce the risk of SCD by addressing modifiable lifestyle factors, especially with a well formulated low-carbohydrate high-fat diet (Lechner et al., 2020); dietary changes can influence health more than exercise itself.

Increased COVID-related SCD appears to be due, at least in part, to a recent history of infection and/or vaccination that induces inflammatory and immune impairment that injures the heart. An unhealthy lifestyle that may include poor diet or overtraining may likely be a contributing factor. The seeming increased incidence of myocarditis and pericarditis during COVID-19 and in the post-vaccination period, and SCD, poses a serious risk to not only athletes but all others and is a cause for alarm. As the population ages and the popularity of running, cycling, and other endurance sports increases, the burden of SCD risk can potentially grow as well. A strong focus on both health and fitness should be a loud and clear public health message.

## Author Contributions

All authors listed have made a substantial, direct, and intellectual contribution to the work and approved it for publication.

## Conflict of Interest

The authors declare that the research was conducted in the absence of any commercial or financial relationships that could be construed as a potential conflict of interest.

## Publisher's Note

All claims expressed in this article are solely those of the authors and do not necessarily represent those of their affiliated organizations, or those of the publisher, the editors and the reviewers. Any product that may be evaluated in this article, or claim that may be made by its manufacturer, is not guaranteed or endorsed by the publisher.
